# Scaling leaf respiration with nitrogen and phosphorus in tropical forests across two continents

**DOI:** 10.1111/nph.13992

**Published:** 2016-05-09

**Authors:** Lucy Rowland, Joana Zaragoza‐Castells, Keith J. Bloomfield, Matthew H. Turnbull, Damien Bonal, Benoit Burban, Norma Salinas, Eric Cosio, Daniel J. Metcalfe, Andrew Ford, Oliver L. Phillips, Owen K. Atkin, Patrick Meir

**Affiliations:** ^1^ School of GeosciencesUniversity of EdinburghEdinburghEH9 3JNUK; ^2^ GeographyCollege of Life and Environmental SciencesUniversity of ExeterAmory BuildingExeterEX4 4RJUK; ^3^ Division of Plant SciencesResearch School of BiologyAustralian National UniversityCanberra2601ACTAustralia; ^4^ Centre for Integrative EcologySchool of Biological SciencesUniversity of CanterburyPrivate Bag4800ChristchurchNew Zealand; ^5^ INRAUMR 1137 Ecologie et Ecophysiologie ForestieresChampenoux54280France; ^6^ INRA UMR‐ECOFOGCampus agronomique ‐ BP 31697379KourouFrench GuianaFrance; ^7^ Environmental Change InstituteSchool of Geography and the EnvironmentUniversity of OxfordSouth Parks RoadOxfordOX1 3QYUK; ^8^ Pontificia Universidad Catolica del PeruSeccion QuimicaAv Universitaria 1801, San MiguelLimaPeru; ^9^ CSIROLand and WaterTropical Forest Research CentreAthertonQLD4883Australia; ^10^ School of GeographyUniversity of LeedsLeedsLS6 2ABUK; ^11^ ARC Centre of Excellence in Plant Energy BiologyDivision of Plant SciencesResearch School of BiologyAustralian National UniversityCanberra2601ACTAustralia

**Keywords:** leaf respiration, nitrogen (N), phosphorus (P), photosynthesis, tropical forest

## Abstract

- Leaf dark respiration (*R*_dark_) represents an important component controlling the carbon balance in tropical forests. Here, we test how nitrogen (N) and phosphorus (P) affect *R*_dark_ and its relationship with photosynthesis using three widely separated tropical forests which differ in soil fertility.
- *R*_dark_ was measured on 431 rainforest canopy trees, from 182 species, in French Guiana, Peru and Australia. The variation in *R*_dark_ was examined in relation to leaf N and P content, leaf structure and maximum photosynthetic rates at ambient and saturating atmospheric CO_2_ concentration.
- We found that the site with the lowest fertility (French Guiana) exhibited greater rates of *R*_dark_ per unit leaf N, P and photosynthesis. The data from Australia, for which there were no phylogenetic overlaps with the samples from the South American sites, yielded the most distinct relationships of *R*_dark_ with the measured leaf traits.
- Our data indicate that no single universal scaling relationship accounts for variation in *R*_dark_ across this large biogeographical space. Variability between sites in the absolute rates of *R*_dark_ and the *R*_dark_ : photosynthesis ratio were driven by variations in N‐ and P‐use efficiency, which were related to both taxonomic and environmental variability.

## Abstract 2

See also the Commentary on this article by McDowell & Xu, **214**: 903–904.

## Introduction

Leaf dark respiration (*R*_dark_) represents a large fraction of total plant respiration (Atkin *et al*., 2007) and, as such, can play an important role in determining the rates of whole‐plant net carbon uptake. In tropical forests, leaf *R*_dark_ comprises a sufficient percentage of total plant respiration (Metcalfe *et al*., 2010; Huntingford *et al*., 2013; da Costa *et al*., 2014; Rowland *et al*., 2014a), such that variations in CO_2_ emissions from *R*_dark_ could determine whether tropical forests act as a source or sink of atmospheric CO_2_ (Meir *et al*., 2008; Gatti *et al*., 2014; Rowland *et al*., 2014a). Consequently, insights into the key determinants of variation in leaf *R*_dark_ are needed to improve estimates of likely shifts in the source and sink capacity of tropical forests under different climate forcing scenarios. In addition to the role of genotype in influencing basal rates of leaf *R*_dark_ (Atkin *et al*., 2015), variations in respiratory fluxes can occur in response to environmental gradients, such as temperature, water availability and nutrient supply (Reich *et al*., 1998a; Meir *et al*., 2001; Wright *et al*., 2006; Atkin *et al*., 2015), and with leaf nitrogen (N) and phosphorus (P) concentrations (Reich *et al*., 1998a; Meir *et al*., 2001; Turnbull *et al*., 2005; Wright *et al*., 2006; Atkin *et al*., 2015). An effect of low leaf nutrient concentration on both leaf *R*_dark_ and photosynthetic capacity has been observed in the tropics, particularly for P (Meir *et al*., 2001, 2007; Kattge *et al*., 2009; Domingues *et al*., 2010; Slot *et al*., 2013, 2014), although the relationships can be complex (Domingues *et al*., 2015) and relatively little is known about the biogeographical variation in leaf *R*_dark_ among tropical forests.

In soil–vegetation–atmosphere modelling frameworks, rates of respiratory CO_2_ release are often associated with leaf photosynthetic CO_2_ uptake (*A*), and leaf physiochemical and/or structural traits (Sitch *et al*., 2003; Medvigy *et al*., 2009; Clark *et al*., 2011). The assumption that leaf *R*_dark_ can be predicted from other traits is supported by a wide range of cross‐biome studies documenting correlations between *R*_dark_, *A*, the maximum rate of carboxylation (*V*_cmax_), leaf N concentration, leaf mass per area (LMA) and leaf lifespan (Ryan, 1995; Reich *et al*., 1997, 1998a,b; Wright *et al*., 2004, 2005, 2006; Atkin *et al*., 2015). However, the variation explained through relationships linking leaf *R*_dark_ to *A*,* V*_cmax_, N and/or LMA is less than the total variation in *R*_dark_ observed in the natural world, given variations among phylogenetically distinct taxa and among differing environments (Reich *et al*., 1998a; Meir *et al*., 2001; Turnbull *et al*., 2003; Wright *et al*., 2006; Atkin *et al*., 2015). Of particular interest for tropical forests is the extent to which gradients in nutrient availability influence *R*_dark_. As respiratory energy is needed for protein turnover in leaves, leaf *R*_dark_ is expected to scale positively with leaf N. However, P limitations are also known to restrict photosynthesis and *R*_dark_ in both temperate and tropical regions (Meir *et al*., 2001, 2007; Turnbull *et al*., 2005; Kattge *et al*., 2009; Domingues *et al*., 2010; Atkin *et al*., 2013). What is less clear is how P limitation, which is commonly observed in tropical forests, might affect *R*_dark_ relationships with *A*, N and LMA.

Like N, P is linked to *R*_dark_ through multiple processes: it is essential for the formation of proteins, nucleic acids and triose phosphate and for the phosphorylation of ADP, and its availability within the leaf can restrict both glycolysis and mitochondrial electron transport (Theodorou *et al*., 1991; Hoefnagel & Wiskich, 1998). Given this, it seems likely that some of the ‘scatter’ in global bivariate relationships linking *R*_dark_ to associated traits could result from regional differences in P availability, including in the tropics, and that *R*_dark_–A, *R*_dark_–N and *R*_dark_–LMA relationships may differ accordingly (Atkin *et al*., 2015). Consequently, in areas of low P, predicting *R*_dark_ using only *A* and/or N‐use efficiency is likely to be insufficient based on the P : N requirements of enzyme synthesis (Domingues *et al*., 2010). Variation in such relationships can also be driven by taxonomy, reflecting unique trait–trait combinations in phylogenetically distinct flora. This may be particularly prevalent in the tropics, where taxonomic diversity is highest (Fyllas *et al*., 2009, Lloyd *et al*., 2010).

Although there is much natural variation in soil and leaf nutrient content across the tropics (Townsend *et al*., 2007; Fyllas *et al*., 2009; Quesada *et al*., 2010), overall it appears that leaf gas exchange is more strongly P‐limited in the tropics relative to many temperate biomes (Meir *et al*., 2001). Tropical forest soils tend to be old and highly weathered, and are therefore more likely to be P‐limited (Quesada *et al*., 2010, 2012). Indeed, in tropical sites in which soil P is low, leaf P has been found to have an influence on *R*_dark_ equal to or greater than that of N (Meir *et al*., 2001, 2007; Domingues *et al*., 2010; Slot *et al*., 2013, 2014); however, this may be strongly moderated by variations in P acquisition by plants from the soil (Gusewell, 2004; Reich & Oleksyn, 2004; Townsend *et al*., 2007). The greater demand for P in photosynthetic, rather than respiratory, pathways suggests that the effects of P limitation are likely to be more pronounced on *A* than on *R*_dark_ (Bloomfield *et al*., 2014). Studies of the effects of P limitation on *R*_dark_ are, however, limited in tropical forests, and studies have yet to fully account for the relative importance of taxonomic and environmental variability among tropical sites on the combined influence of P and N on *R*_dark_.

Here, we examine how leaf N, P and structure affect *R*_dark_ and the *R*_dark_ : *A* ratio at tropical forest sites differing in soil nutrient availability and species composition, with our study contrasting moist tropical rainforests of eastern and western South America (French Guiana and Peru, respectively) with those of Far North Queensland in Australia. French Guiana and Peru provide sites on soils with a strong contrast in N and P availability, with some overlap in floristic composition, whereas the Australian sites have higher soil N and P than French Guiana, but with no floristic overlap with the South American sites. Using this multi‐region dataset, we examine the role of leaf nutrient content and phylogeny in determining *R*_dark_ in tropical forests. In particular, we focus on the possible modulating effects of low leaf P on *R*_dark_, and its relationships with N, LMA and A, using the following hypotheses:

- H1: *R*_dark_ will be lowest at sites with low soil and leaf P concentrations.
- H2: *R*_dark_ at a given leaf N or LMA will be lower where leaf P is more limiting.
- H3: P limitation will be greater on *A* than on *R*_dark_, increasing the *R*_dark_ : *A* ratio at P‐deficient sites.
- H4: Phylogenetic variation will alter the slope and/or elevation of the relationships of *R*_dark_ to *A*, leaf N, leaf P and LMA.

## Materials and Methods

### Sites

The study was carried out at three moist tropical forest sites in: the Paracou research station in French Guiana (FG); Tambopata Biological reserve in the Madre de Dios region of Peru; and multiple sites in Far North Queensland, Australia (AUS). In FG, three permanent plots were inventoried: GX1, GX9 and GX7; however, GX1 and GX9 were considered as a single plot as in Rowland *et al*. (2013, 2014a). In Peru, studies were performed on two permanent plots (TAM‐05 and TAM‐06) of the joint GEM (http://gem.tropicalforests.ox.ac.uk/) and RAINFOR (http://www.geog.leeds.ac.uk/projects/rainfor) projects. Summaries of the vegetation structure, species composition and soils of each plot are given in Tables 1 and 2, and further details can be found in recent literature for FG (Bonal *et al*., 2008; Ferry *et al*., 2010; Rowland *et al*., 2013, 2014a), Peru (Malhi *et al*., 2014; Rowland *et al*., 2014b) and AUS (Torello‐Raventos *et al*., 2013; Weerasinghe *et al*., 2014). FG has a highly seasonal climate: on average, it has the greatest rainfall (Table 1); however, it has a pronounced dry season from August to November when rainfall is often reduced to < 50 mm per month (Bonal *et al*., 2008). At the Peru site, there is a dry season length of 4–5 months (Malhi *et al*., 2014); however, it often receives more rainfall than FG (> 50 mm) in these months (Malhi *et al*., 2014). The Australian plots are located in Far North Queensland: Kauri Creek (KCR‐01); Koombooloomba (KBL‐03); and Cape Tribulation (CTC‐01). Species diversity was lower than that of the South American plots (Torello‐Raventos *et al*., 2013), but differences in species composition between the AUS plots were substantial with no species in common with the Peru or FG sites. Within our dataset, the three most common tree families in FG, Peru and AUS and their proportions of all trees in our samples are as follows: FG – Lecythidaceae (12%), Caesalpiniaceae (11%) and Chrysobalanaceae (9%); Peru – Moraceae (10%), Violaceae (7%) and Myristicaceae (6%); AUS – Lauraceae (28%), Elaeocarpaceae (8%) and Proteaceae (8%). Part of our data contributed to the global analysis of Atkin *et al*. (2015), but this regional analysis is new, includes more datasets and yields new insights.

**Table 1:** Description of site, country, location, climate and soil type for the plots used in this study

Site	Country	Latitude	Longitude	Elevation (m asl)	MAP (mm)	MAT (°C)	WRB soil classification
Paracou (GX1 & GX9)	French Guiana	5.28°N	−52.92°W	≈ 40	3041	25.8	Acrisols
Paracou (GX7)	French Guiana	5.27°N	−52.91°W	≈ 10	3041	25.8	Gleysoils
Tambopata plot 3 (TAM‐05)	Peru	−12.83°S	−69.27°W	220	2463	25.53	Haplic Cambisol
Tambopata plot 4 (TAM‐06)	Peru	−12.84°S	−69.30°W	200	2463	25.63	Haplic Alisol
Kauri Creek (KCR‐01)	Australia	−17.11°S	145.60°E	813	1960	20.5	Haplic Cambisol
Koombooloomba (KBL‐03)	Australia	−17.68°S	145.53°E	1055	1340	19.1	Haplic Nitisol
Cape Tribulation (CTC‐01)	Australia	−16.10°S	145.45°E	90	3200	25.2	Haplic Cambisol

Data on plot elevation in metres above sea level (asl), mean annual precipitation (MAP) and mean annual temperature (MAT) are shown. Soil status follows the World Reference Base classification. Peru soil and climate descriptions are reported in Quesada *et al*. (2010) and Malhi *et al*. (2014). Details for French Guiana soils and climate are reported in Ferry *et al*. (2010) and Bonal *et al*. (2008). Details of the Australian soils and climate are reported in Weerasinghe *et al*. (2014).

**Table 2:** Soil texture and chemistry for each plot used in this study

Site	Country	Soil texture			Soil chemistry						
		Clay	Sand	Silt	C	N	C : N	P_Total_	P_Olsen_	CEC	pH
		Fraction			(g kg^−1^)	(g kg^−1^)	ratio	(mg kg^−1^)	(mg kg^−1^)	(mmol kg^−1^)	
Paracou (GX1 & GX9)	FG	0.43	0.48	0.09	30.9	1.9	16.2	276.0	4.4	27.6	4.6
Paracou (GX7)	FG	0.33	0.57	0.10	22.5	1.4	15.8	170.0	8.0	20.5	4.7
Tambopata plot 3 (TAM‐05)	Peru	0.44	0.40	0.17	15.1	1.6	9.4	256.3	11.8	44.7	3.9
Tambopata plot 4 (TAM‐06)	Peru	0.46	0.02	0.52	12.0	1.7	7.1	528.8	11.7	56.7	5.1
Kauri Creek (KCR‐01)	AUS	0.20	0.55	0.25	38.9	2.9	20.3	345.9	34.0	23.6	5.4
Koombooloomba (KBL‐03)	AUS	0.32	0.22	0.46	40.7	2.6	15.3	292.1	NA	8.6	4.4
Cape Tribulation (CTC‐01)	AUS	0.28	0.19	0.54	35.8	3.0	16.9	473.1	15.8	27.4	5.6

Nutrient levels are shown for carbon (C), nitrogen (N) and phosphorus (P). Phosphorus is reported in two forms – total and Olsen. For comparative purposes, P_Olsen_ is taken as the sum of the resin and bicarbonate inorganic fractions. Cation exchange capacity (CEC) performed at soil pH is the summation of exchangeable Ca, Mg, K, Na and Al (Quesada *et al*., 2010; Ferry *et al*., 2010; Weerasinghe *et al*., 2014; V. Freycon, pers. comm.). FG, French Guiana; AUS, Australia.

The different plots represent an overall gradient in soil P fertility from least fertile in FG, where P has been considered to be particularly limiting (Baraloto *et al*., 2005; Ferry *et al*., 2010), to more fertile plots in Peru and AUS.

### Leaf sampling and gas exchange measurements

At each site, data were collected following the end of the wet season: May 2009 in AUS; May–July 2010 in Peru; and September–November 2010 in FG. Trees were selected according to the following criteria. First, the trees should be dominant or co‐dominant in the canopy, so that a major proportion of their leaves would be exposed to full sunlight for much of the day. Second, a large range of species was sampled at each site in order to sample a wide range of leaf traits. Third, species were selected to include the most abundant local species. Fourth, the species which were found to be in common between the FG and Peru sites were prioritized. Finally, at the two South American sites, among the list of target species, those trees which were clustered were selected so as to optimize canopy branch sampling by tree climbers. For two of the AUS plots, equivalent branches were pulled down using a weighted line shot from a catapult (KCR‐01 and KBL‐03), whereas a 48‐m tall industrial crane provided access to the canopy at CTC‐01. With some noted exceptions, little replication of individual species was possible at most sites. Each tree was initially identified to the genus level and, whenever possible, to the species level. We prioritized the sampling of as many trees as possible, and therefore only sampled one leaf per tree; 431 leaves were sampled across sites from 182 species.

Detached branches were immediately re‐cut under water to restore hydraulic connectivity; this method has been found previously not to affect leaf *R*_dark_ (Turnbull *et al*., 2003; Cavaleri *et al*., 2008; Rowland *et al*., 2015). However, we also tested this assumption on a subset of attached and then detached branches at the sites, with no impact of branch removal discernible in our comparisons (*n* = 20, *P* > 0.05; Supporting Information Fig. S1). Cutting effects on photosynthetic capacity measurements have also been found to be negligible elsewhere in the tropics (Rowland *et al*., 2015); however, we acknowledge that cutting effects have been found in other studies (Santiago & Mulkey, 2003). If cutting effects did exist on photosynthetic measurements here, they would have been minimized through the correction of all photosynthetic values to a common internal CO_2_ concentration and temperature (Eqn 1).

All gas exchange measurements were made between 08:00 h and 17:00 h; instantaneous measurements of light‐saturated photosynthesis (*A*_sat_) and of leaf respiration in darkness (*R*_dark_) were made using a Li‐Cor 6400 portable photosynthesis system (Li‐Cor Inc., Lincoln, NE, USA). Measurements were conducted with the leaf chamber block temperature set to 25–28°C (close to ambient temperature). Air flow rate through the chamber was set to 300–500 μmol s^−1^ during photosynthesis measurements and to 300 μmol s^−1^ during *R*_dark_ measurements.

Light‐saturated (2000 μmol photons m^−2^ s^−1^) photosynthetic data were obtained at ambient atmospheric CO_2_ (400 ppm) at all sites, denoted here as *A*_sat_. In all cases, measurements were conducted at a relative humidity of *c*. 70% and after the leaves had been exposed to saturating irradiance in the chamber for 10 min. Following completion of photosynthesis measurements, leaves were darkened for 30 min to ensure that steady‐state conditions had been achieved (Azcon‐Bieto & Osmond, 1983; Atkin *et al*., 1998). Note: using a subset of leaves, we also tested the effect of darkness period on the *R*_dark_ measurement, recording gas exchange data at 1‐min intervals after fully darkening each leaf to make sure we avoided any post‐illumination burst in our subsequent measurements of leaf respiration. Our data (not shown) indicated that reliable *R*_dark_ measurements were possible only following a minimum of 20–25 min of darkness. To test the effect of time of day on *R*_dark_, measurements were made at dawn, dusk and at regular intervals during the day, on a subset of leaves (*n *= 9; three species, three leaves per species, Fig. S2). To enable comparison of fluxes at a common temperature, *R*_dark_ was corrected to 25°C using Eqn 1 (Atkin & Tjoelker, 2003):

(Eqn 1) $$R_{\mathrm{dark}25}=R_{\mathrm{dark}}\cdot {Q_{10}}^{(\frac{25-T_{\mathrm{leaf}}}{10})}$$

where *R*_dark25_ is the rate calculated at the reference temperature, in our case 25°C, Q_10_ is 2.2 (Meir *et al*., 2001; Atkin *et al*., 2005; Rowland *et al*., 2015) and *R*_dark_ is the rate measured at ambient leaf temperature, *T*_leaf_. Given the effect of temperature and variations in stomatal conductance on photosynthesis and internal CO_2_ concentrations, photosynthesis rates were also corrected to 25°C and to a common internal CO_2_ concentration, *C*_i_, being the median *C*_i_ values measured for all *A*_sat_ measurements made across all sites (270 ppm). The derived *R*_dark_ and *C*_i_ values were used in the Farquhar, von Caemmerer and Berry model of photosynthesis (Farquhar *et al*., 1980) to calculate standardized *A*_sat_ values according to:

(Eqn 2) $$A_{\mathrm{sat}\_ci\_25^{\circ }C}=\left[\frac{V_{\mathrm{cmax}{}_{25}}\left(Ci_{270}-\Gamma *\right)}{Ci_{270}+\left(K_{c}*\left(1+\frac{210}{K_{o}}\right)\right)}\right]-R_{\mathrm{dark}25}$$

where *V*_cmax25_ represents the maximum rate of carboxylation, Γ* is the CO_2_ compensation point, *K*_c_ and *K*_o_ represent the Michaelis–Menton constants for the carboxylase and oxygenase enzymes, respectively, and ‘25’ denotes metabolic fluxes temperature corrected to 25°C. Γ*, *K*_c_ and *K*_o_ were scaled to leaf temperature and thus calculated per leaf sample following Farquhar *et al*. (1980). *V*_cmax_ at the prevailing leaf temperature (at the time of measurement, *V*_cmax_*t*_) was calculated according to:

(Eqn 3) $$\begin{array}{cc} & V_{\mathrm{cmax}\_t}=\frac{\left(A_{\mathrm{sat}{}_{t}}+R_{\mathrm{dark}{}_{t}}\right)(C_{i_{t}}+\left(K_{c}*\left(1+\frac{210}{K_{o}}\right)\right)}{\left(C_{i\_t}-\Gamma *\right)} \end{array}$$

where ‘*t*’ denotes values at the time of measurement. *V*_cmax_*t*_ was corrected to 25°C following Sharkey *et al*. (2007).

### Leaf structural traits and chemical composition

Following gas exchange, leaves were detached and stored in a re‐sealable plastic bag containing a piece of damp paper tissue. Once in the laboratory, the leaf surface was dried and scanned to enable subsequent calculation of leaf area using ImageJ software (http://rsbweb.nih.gov/ij/), and then oven dried at 60°C to constant mass. Subsequently, dry mass was recorded and leaf samples were ground in a ball mill and analysed for carbon (C), N and P content. The mass and area data were used to determine ratios of LMA.

Leaf N content was determined by elemental analysis (EURO EA 3000 series, Eurovector Elemental Analyser for CHNS‐O; EuroVector SpA, Milan, Italy). Leaf P content was determined as described by Lloyd *et al*. (2009), using inductively coupled plasma optical emissions spectrometry (ICP‐OMS; PerkinElmer Optima 5300DV, PerkinElmer, Shelton, CT, USA), after a nitric–perchloric acid digestion (MAFF 1986; Method no. 41, RF427).

### Data analysis

All analysis was performed in the statistical package R (R.2.14.2, R‐project software, http://www.r-project.org). As a result of the possibility of error when measuring leaf traits, we chose to eliminate outliers from our dataset; all trees which had data for log‐transformed N, P, LMA, specific leaf area (SLA), *R*_dark_ or *A*_sat_ which were more than three standard deviations from the mean were eliminated from our dataset (22 trees; 4.9% of the dataset). Following the exclusion of outliers, we analysed data from 232 trees in Peru, 141 trees in FG and 58 trees in AUS.

Standardized major axis (SMA) regression was used to test for variations in the slope and elevation of bivariate leaf trait relationships between the three sites. SMA regression analysis assumes a normal data distribution and, therefore, following initial inspection of the data using the qqnorm function in R, all of our data for SMA regression analysis were log transformed. Bivariate analyses do not account for the likely effects of co‐limitation or phylogenetic variation on *R*_dark_, both of which are likely to limit the predictive power of any single relationship of N, P, LMA and SLA with *R*_dark_ To account for this and to identify the parameters with the most predictive power for modelling *R*_dark_, we used mixed‐effect modelling. We included N, P and LMA or SLA as fixed effects, and plots and tree species nested within plots as random effects. Multiple models were compared in a procedure including both fixed and random effects; Akaike's Information Criteria (AICs) were used to compare models with the aim of simplifying the preferred model to its most parsimonious form. Data were not log transformed for the mixed‐effect modelling analysis. Inter‐site differences were tested with non‐parametric Wilcoxon tests.

Given recent debates about the relative merits of leaf traits expressed on an area and a mass basis (Lloyd *et al*., 2013; Osnas *et al*., 2013; Poorter *et al*., 2014), we chose to present our results on both a mass and an area basis.

## Results

**Figure 1:**
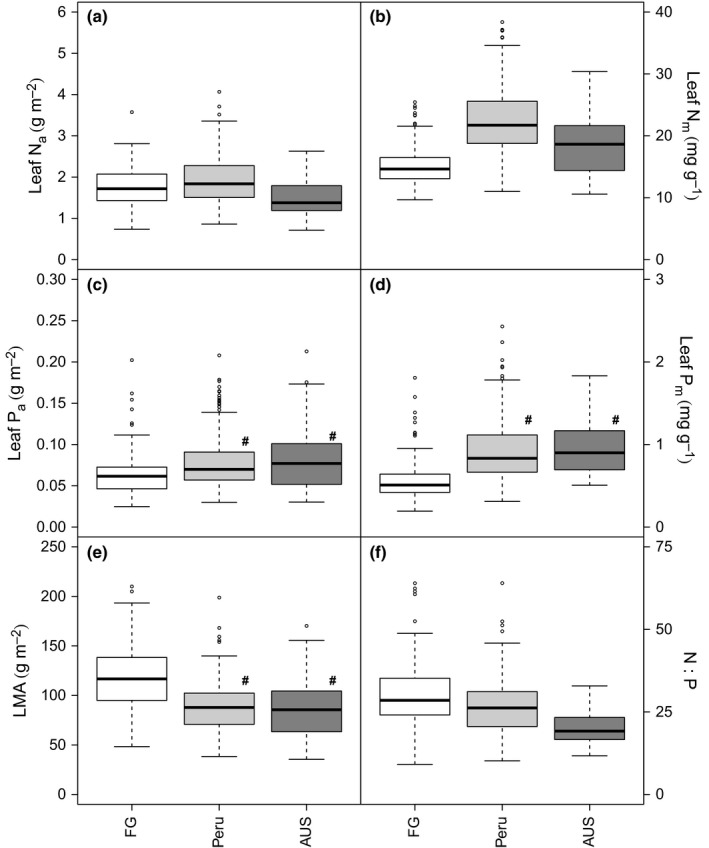
Boxplots of leaf nitrogen (N) on an area (a) and mass (b) basis, leaf phosphorus (P) on an area (c) and mass (d) basis, leaf mass per area (LMA, e) and leaf N to P ratio (N : P, f). Pairwise Wilcoxon tests were performed and site means which were not significantly different at the *P *< 0.05 level are shown by #. The thick line shows the median, the box extends to the lower and upper quartiles, the dashed lines indicate the nominal range (1.5 times the interquartile range below and above the upper and lower quartiles) and the circles indicate points which lie outside of the nominal range.

Leaves sampled in FG exhibited the lowest mass‐based values of leaf N (median = 14.6 mg N g^−1^) and P (median = 0.5 g P g^−1^). The nutrient content of FG leaves was significantly lower than that of leaves sampled in Peru and AUS, on both an area and a mass basis for P (P_a_, *P *< 0.002; P_m_, *P *< 0.001; Fig. 1c,d) and on a mass basis for N (N_m_, *P *< 0.001; Fig. 1b). FG leaves also exhibited the highest LMA (Fig. 1e) and N : P ratio (Fig. 1f). Leaves from Peru had the highest leaf N levels on an area and mass basis (Fig. 1a,b). Peru and AUS leaves exhibited similar values for leaf P and LMA; however, AUS leaves had the lowest N : P ratio (Fig. 1f).

**Figure 2:**
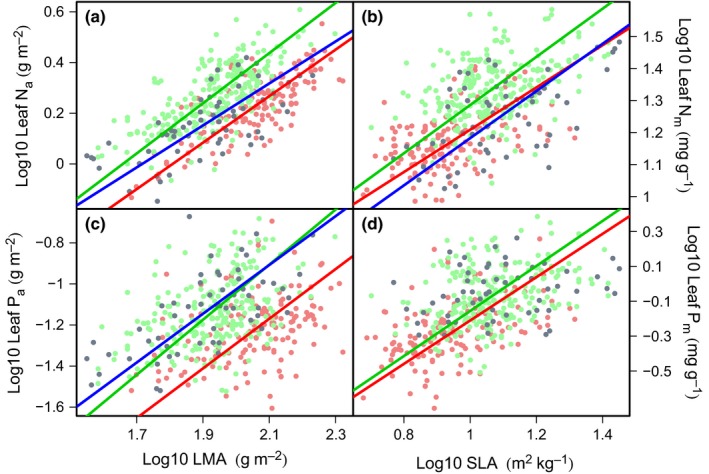
Log–log plots of leaf mass per area (LMA) against leaf phosphorus (P; c) and nitrogen (N; a) on an area basis and specific leaf area (SLA) against leaf P (d) and leaf N (b) on a mass basis. Data for individual leaves are shown as points separated by country: green (Peru), red (French Guiana) and blue (Australia). Standardized major axis (SMA) lines are shown for the relationships of each country. Tests for significant differences in the slope and *y*‐axis of the SMA lines are shown in Table 3. Note: if the linear relationship between variables is not significant, SMA lines are not shown.

Area‐based values of N and P scaled positively with LMA; the same was true for mass‐based N and P relationships with SLA (Fig. 2a–d). There were no significant differences in the slopes of these relationships among the three countries (Fig. 2a–d; Table 3). However, there were significant differences in the elevations of the relationships, with FG leaves typically exhibiting lower nutrient values at any given LMA or SLA value than leaves from Peru (Fig. 2a–d; Table 3). Across all countries, the relationships of N_a_ and P_a_ to LMA were stronger than N_m_ and P_m_ to SLA (Table 3).

**Table 3:**
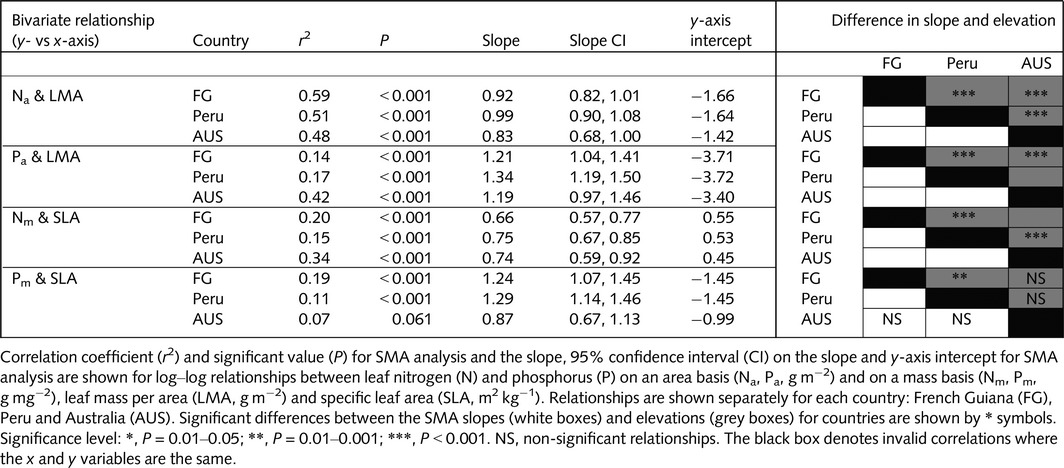
Results for standardized major axis (SMA) regression analysis of relationships between leaf structure and leaf nutrient content

**Figure 3:**
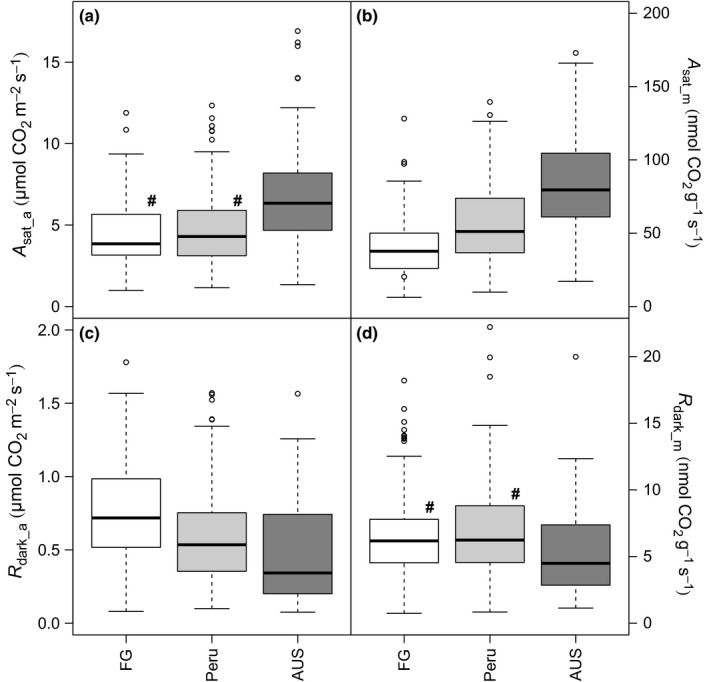
Boxplots of saturating photosynthesis on an area (*A*_sat_a_; a) and mass (*A*_sat_m_; b) basis and respiration in the dark on an area (*R*_dark_a_; c) and mass (*R*_dark_m_; d) basis. Pairwise Wilcoxon tests were performed and datasets which were not significantly different at the *P *< 0.05 level are shown by #. The thick line shows the median, the box extends to the lower and upper quartiles, the dashed lines indicate the nominal range (1.5 times the interquartile range below and above the upper and lower quartiles) and the circles indicate points which lie outside of the nominal range. FG, French Guiana; AUS, Australia.

**Figure 4:**
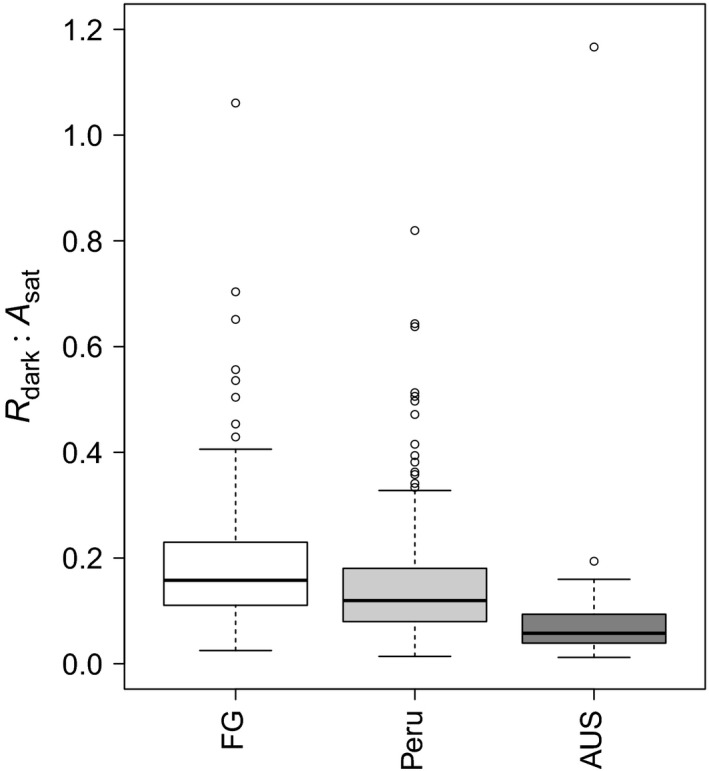
Boxplots of respiration in the dark divided by saturating photosynthesis (*R*_dark_ : *A*_sat_). Pairwise Wilcoxon tests were performed and all datasets were significantly different at the *P *< 0.05 level. The thick line shows the median, the box extends to the lower and upper quartiles, the dashed lines indicate the nominal range (1.5 times the interquartile range below and above the upper and lower quartiles) and the circles indicate points which lie outside of the nominal range. FG, French Guiana; AUS, Australia.

Overall, the rates of light‐saturated photosynthesis (*A*_sat_) at a common *C*_i_ and temperature were higher in AUS (on both an area and mass basis; Fig. 3a,b) compared with leaves sampled in Peru and FG. On an area basis, FG and Peru exhibited similar rates of *A*_sat_, whereas, on a dry mass basis, *A*_sat_ was significantly lower in FG than Peru linked to higher LMA for the FG leaves. *R*_dark_a_ was significantly higher in FG than Peru, with rates also being higher in Peru than in AUS (Fig. 3c); however, expressed on a mass basis, rates of *R*_dark_m_ were generally similar in FG and Peru, but lower in AUS (Fig. 3d). Higher photosynthesis and lower respiration in AUS relative to the South American countries resulted in low *R*_dark_ : *A*_sat_ ratios (Fig. 4), with *R*_dark_ : *A*_sat_ being greater in FG than in Peru (reflecting low rates of *A*_sat_ and high rates of *R*_dark_ in FG). Thus, overall, the foliar carbon processing capacities of leaves were more ‘favourable’ in AUS than in the two South American sites, reflecting higher rates of photosynthetic CO_2_ uptake and lower or similar rates of respiratory CO_2_ release, with the balance being least favourable in leaves from FG (which also exhibited the lowest leaf P concentrations; Fig. 1d).

**Figure 5:**
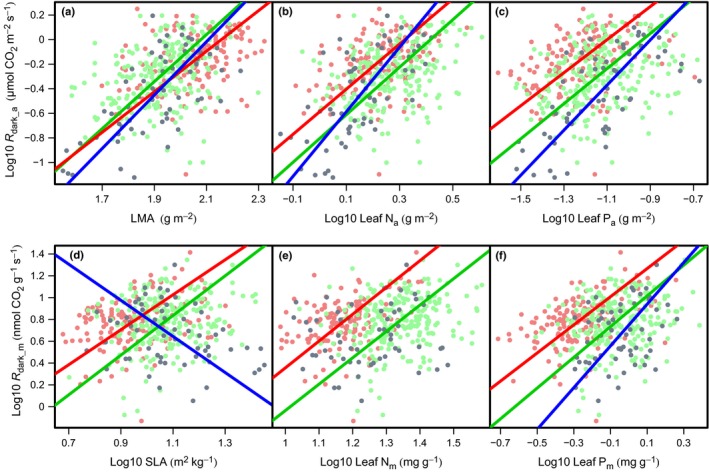
Log–log plots of respiration in the dark on an area basis (*R*_dark_a_) against leaf mass per area (LMA; a), leaf nitrogen (N_a_; b) and leaf phosphorus (P_a_; c), and respiration in the dark on a mass basis (*R*_dark_m_) against specific leaf area (SLA; d), leaf nitrogen (N_m_; e) and leaf phosphorus (P_m_; f). Data for individual leaves are shown as points separated by country: green (Peru), red (French Guiana) and blue (Australia). Standardized major axis (SMA) lines are shown for the relationships of each country. Tests for significant differences in the slope and *y*‐axis of the SMA lines are shown in Table 4. Note: if the linear relationship between variables is not significant, SMA lines are not shown.

**Figure 6:**
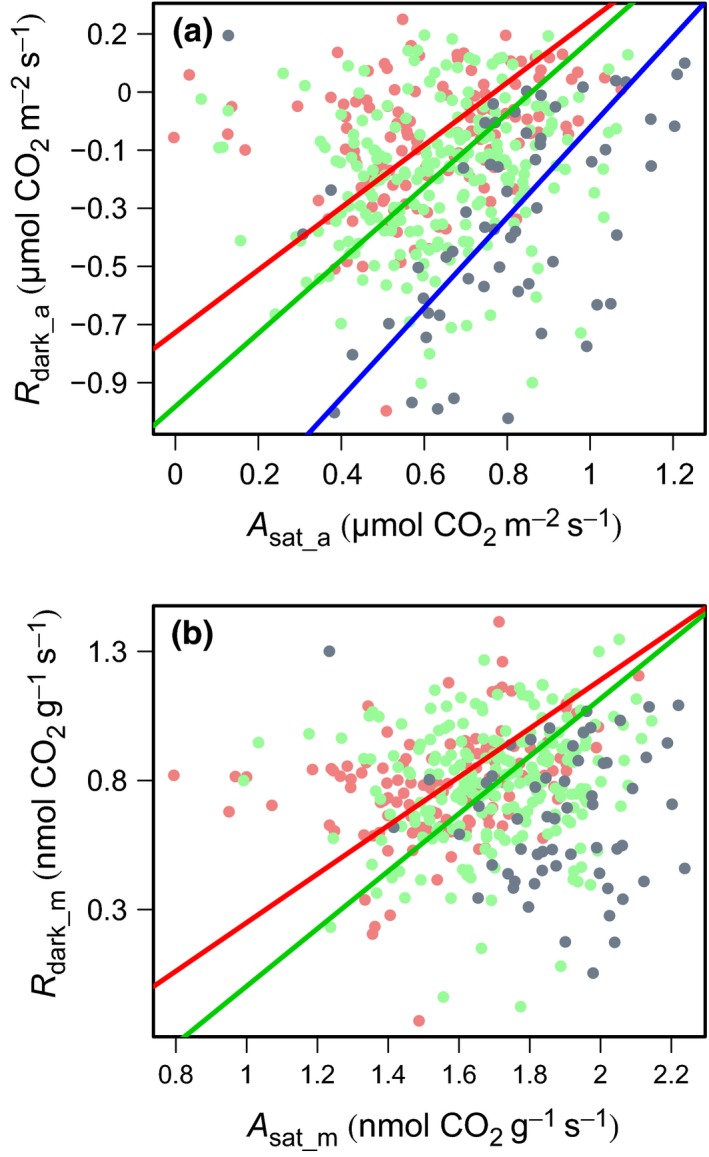
Log–log plots of respiration in the dark on an area basis (*R*_dark___a_) against saturating photosynthesis on an area basis (*A*_sat_a_) (a) and respiration in the dark on a mass basis (*R*_dark___m_) against saturating photosynthesis on a mass basis (*A*_sat_m_) (b). Data for individual leaves are shown as points separated by country: green (Peru), red (French Guiana) and blue (Australia). Standardized major axis (SMA) lines are shown for the relationships of each country. Tests for significant differences in the slope and *y*‐axis of the SMA lines are shown in Table 5. Note: if the linear relationship between variables is not significant, SMA lines are not shown.

Across all three countries, *R*_dark_ scaled positively with N, P and *A*_sat_ on an area basis, and with LMA; *R*_dark_ also scaled positively with N, P and *A*_sat_ on a mass basis, and with SLA (Figs 5, 6); however, the relationships were consistently stronger on an area basis than on a mass basis (Table 4). The strength of the relationships may vary considerably between sites, with *R* ^2^ values being considerably higher in AUS than at the South America sites. At the South American sites, no strong relationships (*R* ^2^ > 0.2) were found. Importantly, the slope and/or elevation of *R*_dark_ relationships often differed among the three countries. Although FG and Peru displayed no significant differences in the slope of the relationships between *R*_dark_ and leaf traits (Fig. 5; Table 4), rates of *R*_dark_ were significantly higher in FG at any given P, N, LMA or SLA value than in Peru (Fig. 5; Table 4; i.e. higher elevations for FG). By contrast, AUS exhibited a significantly different slope to both FG and Peru for the *R*_dark_ to P relationships on both an area and mass basis, and for the *R*_dark_ to N relationship on an area basis (Fig. 5b,c,f; Table 4), and it showed a significantly steeper *R*_dark_a_–LMA slope than FG (Fig. 5a; Table 4).

**Table 4:**
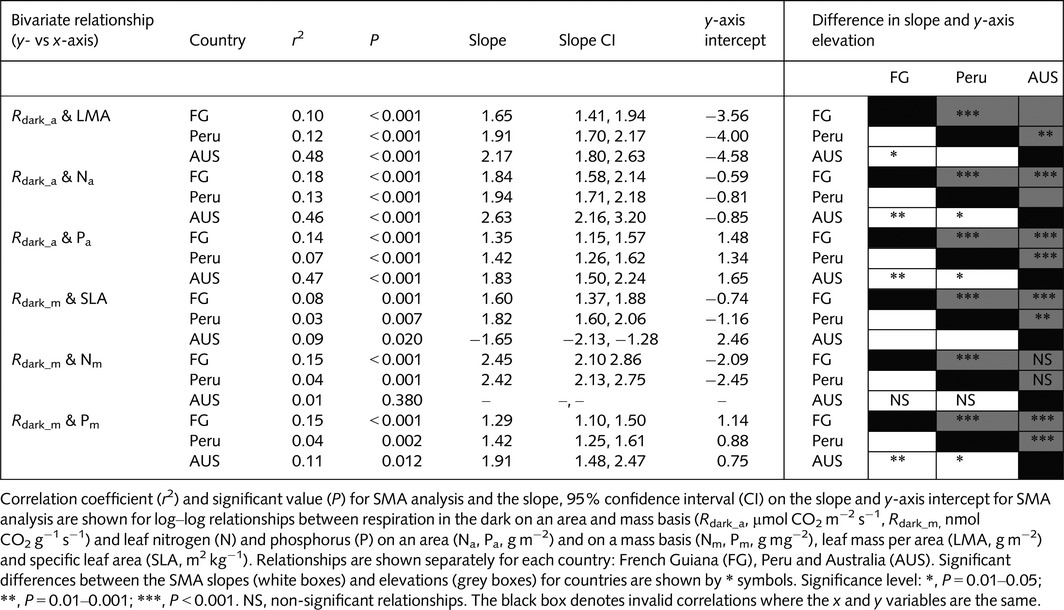
Results of standardized major axis (SMA) regression analysis of *R*_dark_ with leaf structure and nutrient content

**Figure 7:**
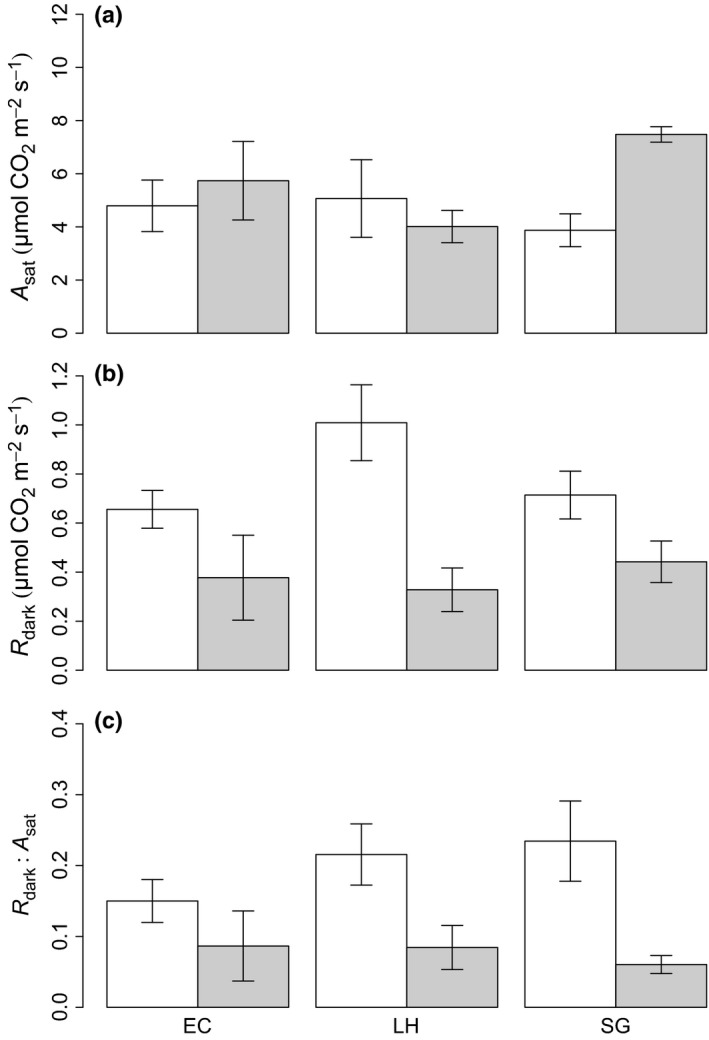
Bar plot showing the mean ± SE for light‐saturated photosynthesis (*A*_sat_; a), leaf respiration in darkness (*R*_dark_; b) and *R*_dark_ divided by *A*_sat_ (c) on an area basis for species common to French Guiana (FG; white bars) and Peru (grey bars): *Licania heteromorpha* (LH), *Eschweilera coriacea* (EC) and *Symphonia globulifera* (SG).

The *R*_dark_–*A*_sat_ relationships for the three sites all showed significantly different slopes and elevations (Fig. 6; Table 5), with FG leaves exhibiting higher rates of *R*_dark_ at low rates of *A*_sat_, compared with leaves sampled in Peru and AUS. As *A*_sat_ was standardized to a constant *C*_*i*_ and temperature, Fig. 6 implies that, at a given *V*_cmax25_, the leaves have significantly different respiration rates among sites. Thus, the respiratory cost per unit photosynthetic capacity is significantly larger at FG than at the other two sites, nearly twice that found for AUS (Fig. 6). Figure 7 demonstrates that species common to both Peru and FG (*Eschweilera coriacea*,* Licania heteromorpha*,* Symphonia globulifera*) had consistently higher *R*_dark_, and thus elevated *R*_dark_ : *A*_sat_ ratios at FG, but no consistent differences in *A*_sat_. As a result of low replication, statistical tests did not show significant differences between the *R*_dark_, *A*_sat_ and *R*_dark_ : *A*_sat_ values for individual species sampled in FG and Peru. However, when the three species were combined, there was a significant difference between *R*_dark_ and the *R*_dark_ : *A*_sat_ ratio between the two countries, being greater in FG (0.79 ± 0.11, *P* < 0.001 and 0.20 ± 0.03, *P* < 0.001, respectively) than in Peru (0.38 ± 0.03, *P* < 0.001 and 0.08 ± 0.01, respectively). This result suggests that, notwithstanding the small sample of species common to both sites, the patterns observed in Fig. 7 appear to hold when controlling for phylogeny.

**Table 5:**
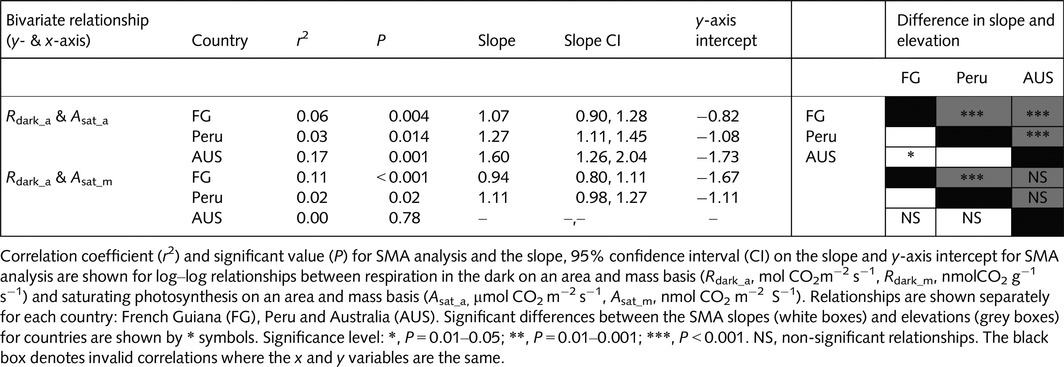
Results of the standardized major axis (SMA) regression analysis of *R*_dark_ with *A*_sat_

Mixed‐effect models provide a means to test which combinations of N, P and LMA or SLA are the best predictors of *R*_dark_ and how this may vary among countries once we take into account the random, unmeasured, influences of environmental and phenotypic variability between plots on *R*_dark_. N and P proved to be important fixed effects for modelling *R*_dark_, on both an area and mass basis, when data from all countries, or from just the South American countries, were combined. However, a large proportion of the variance in the data could be attributed to the random variables; species nested within plot and plot alone accounted for 30–33% of the variance for both the universal and South American models on mass and area bases (Table 6). In the country‐by‐country models, for FG and Peru, the random effects of species and plot explained a lower proportion of the variance in the data (Table 6). In AUS, there were limited effects of species on the model of *R*_dark_, but substantially larger plot effects. In FG, the country with the lowest N and P (Fig. 1c,d), both N and P were important fixed effects for explaining *R*_dark_ on both an area and mass basis (Table 6). By contrast, for Peru and AUS, where levels of foliar P are higher than in FG, LMA and SLA were of greater importance and the importance of N or P varied depending on whether terms were expressed on an area or mass basis (Table 6).

**Table 6:** Results of the mixed‐effect model analysis of *R*_dark_

	Linear data to MEM comparison			Random variance (%)		
	Best model	*r* ^2^	*P*	Plot : species	Plot	Residual
Area basis						
All	LMA + N + P	0.55	< 0.01	18.58	12.52	68.90
South	N × P	0.55	< 0.01	19.71	13.13	67.16
FG	N + P	0.40	< 0.01	11.29	0.51	88.20
Peru	LMA + N	0.55	< 0.01	26.93	0.00	73.07
AUS	LMA + P	0.61	< 0.01	6.87	31.69	61.44
Mass basis						
All	N + P	0.51	< 0.01	19.25	13.35	67.40
South	SLA × N × P	0.51	< 0.01	19.86	10.30	69.84
FG	N × P	0.43	< 0.01	5.44	0.00	94.56
Peru	SLA + P	0.51	< 0.01	23.17	2.92	73.91
AUS	SLA × N	0.51	< 0.01	0.00	39.34	60.66

Mixed‐effect model (MEM) results show which combination of nitrogen (N), phosphorus (P), leaf mass per area (LMA) and specific leaf area (SLA) provides the best model for leaf respiration in the dark *R*_dark_ on an area and mass basis. Species nested within plot was used as a random component of the model. The best model for *R*_dark_ for all countries (All), South American countries (South) and for French Guiana (FG), Peru and Australia (AUS) are shown. The coefficient of variation (*r* ^2^) and significance (*P*) of the linear regression of the modelled vs measured data are also shown and the contribution of each random effect to the variance with the dataset.

## Discussion

We observed significant variations in average leaf N and P among FG, Peru and AUS, with FG having the lowest leaf P_m_ and N_m_, and the highest leaf N : P ratio (Fig. 1), which translated directly into biogeographical differences in the relationships between N, P and LMA or SLA and *R*_dark_. In particular, our results demonstrate: (1) the importance of leaf P in accounting for variation in *R*_dark_ across three tropical regions with markedly different soil and foliar P levels; (2) the importance of leaf N in contributing to variations in leaf *R*_dark_; and (3) the differing relative influence of each nutrient on the variation in *R*_dark_ among the three countries. Our results show that, within tropical forests, rates of *R*_dark_ per unit leaf area, and leaf N and P mass, are greater at sites with plots containing the lowest total soil P (222.5 mg kg^−1^ average at FG compared with 392.6 and 370.4 mg kg^−1^ for Peru and AUS, respectively; Table 2). Similarly, the ratio of *R*_dark_ : *A* was largest at the site with the smallest leaf P. Importantly, the elevated *R*_dark_ at the sites with low leaf P was not matched by significant changes in *A*_sat_, suggesting less sensitivity in *A*_sat_ than *R*_dark_ to P at the leaf concentrations observed. These results are of direct relevance to the modelling of the carbon cycle in tropical forests, as most models assume that leaf nutrient limitations affect *A* and *R*_dark_ in equal measure, with both fluxes restricted in proportion by decreasing leaf nutrient availability.

### Relationships between *R*_dark_ and leaf traits

Work across a range of biomes has suggested that levels of foliar N and P are important predictors of leaf gas exchange and may limit *R*_dark_ and photosynthetic fluxes differently (Reich *et al*., 1998a, 2009; Turnbull *et al*., 2005; Alvarez‐Clare *et al*., 2013; Atkin *et al*., 2015). In particular, leaf P is thought to limit both leaf photosynthetic and respiratory fluxes more than leaf N in tropical forests (Meir *et al*., 2001; Domingues *et al*., 2010, 2015; Alvarez‐Clare *et al*., 2013). The results of the bivariate relationships of N, P, LMA and SLA with *R*_dark_, although highly significant, were weak, particularly at the South American sites. This is most likely because multiple‐linear models often provide a far better representation of *R*_dark_ (Reich *et al*., 1998a; Meir *et al*., 2001; Slot *et al*., 2014) through being able to account for the effects of co‐limitation. The mixed‐effect modelling analysis performed here demonstrated that, when modelling data from all countries, both N and P were consistently significant as variables for predicting *R*_dark_ (Table 6), on both an area and mass basis. However, we note with caution that the model combining data from all countries is likely to be biased towards South America as our AUS sample contained fewer trees (58) than Peru (232) and FG (141). The final preferred combination of the variables N, P and LMA or SLA for modelling *R*_dark_ varied on a country‐by‐country basis, with N and P combined being most important predictors of *R*_dark_ in FG, the country with the lowest leaf nutrient concentration (Fig. 1). These regionally dependent differences in the preferred mixed‐effect model structure were consistent with the observed variation in the *R*_dark_–trait relationships from the SMA regression analysis.

The slopes and strengths of the relationships of key leaf traits (N, P and LMA or SLA) with *R*_dark_ showed significant biogeographical variation. Contrary to our first and second hypotheses, within the South American sites, FG, the site with the lowest leaf and soil P, maintained significantly greater absolute *R*_dark_ values and *R*_dark_ at any given value of N or P (Figs 3, 5). This difference suggests that, for a given leaf nutrient investment, there is a larger *R*_dark_ cost in FG leaves relative to those in Peru (Fig. 5), just as FG leaves have a greater LMA per unit N or P relative to Peru (Fig. 2). These shifts could be caused by differences in nutrient allocation to metabolism vs structure. N is lower on an area, but not mass, basis in FG compared with Peru and AUS (Fig. 2b), suggesting differences in the allocation of N to leaf structure in FG with respect to Peru and AUS, which could be driven by varying environment and/or taxonomy. If there is a fundamental minimum leaf N and P required per unit of base respiration at all sites (De Vries, 1975; Amthor, 1989), the elevated *R*_dark_ per unit N or P at FG (Fig. 5) may suggest that proportionally more of the total leaf N and leaf P is invested in *R*_dark_ (i.e. higher respiratory enzyme capacity) at FG than is invested at the other sites. Alternatively, respiratory enzyme capacity may be constant across sites, but with demand for respiratory products being greater at FG than at the other sites, perhaps reflecting other environmental factors, such as regional differences in aridity (Metcalfe *et al*., 2010; Atkin *et al*., 2015; Rowland *et al*., 2015), limitations in other (unmeasured) nutrients or a more complex co‐limitation of nutrients that may interact with other factors, such as plant life history (Townsend *et al*., 2007; Alvarez‐Clare *et al*., 2013).

Although the South American sites maintained similar slopes across *R*_dark_–leaf trait relationships, these slopes differed significantly from those found in AUS (Table 4; Fig. 5). The significant differences in slope of the *R*_dark_–trait relationships for the Australian sites suggest that the biological processes that determine how *R*_dark_ varies with increasing nutrient availability vary across continents. This may result from the Australian sites being taxonomically distinct to the South American sites. Similarly, it may reflect other environmental variations among the sites, including an average difference in mean annual temperature (MAT) of up to 7°C between the Australian and South American sites (Table 1). Such a variation in mean annual growing temperature has been shown to significantly influence leaf respiration rates in global studies (Atkin *et al*., 2015) and could drive a shift in how *R*_dark_ changes with increasing nutrient availability, although there was insufficient power in our data to test this in our study. We suggest that these climatic limitations, as well as phylogenetic limitations and the potential for interactive effects and co‐limitation of N, P, LMA and SLA, can explain why the predictive power for *R*_dark_ achieved within the mixed‐effect modelling analysis (Table 6) is greater than that of the bivariate SMA relationships (Table 4).

### Relationships between *R*_dark_ and photosynthesis

The relationship between *R*_dark_ and *A* is not constant and would be expected to vary in situations in which environmental stresses, such as high temperature, drought or nutrient limitation, have a differential impact on *R*_dark_ and *A* (Reich *et al*., 1998a; Meir *et al*., 2001; Atkin *et al*., 2008, 2015; Atkin & Macherel, 2009; Domingues *et al*., 2010, 2015). In this study, we demonstrated that tropical rain forest leaves from biogeographically distinct countries, which have different nutrient contents and experience differences in seasonal water limitation, also vary in how *R*_dark_ scales with *A* (Fig. 6). Although we indeed found that the *R*_dark_ : *A* ratio was highest at the most P‐deficient site (FG), this was not caused by the hypothesized effects of lower leaf P having a greater impact on *A* than *R*_dark_; rather, it primarily reflected rates of leaf *R*_dark_ being greater at the low‐P sites (with *A* constant across the two South American countries). Our results also suggest that this result is not significantly confounded by taxonomic differences between the sites, as the direct comparison of species common to FG and Peru demonstrated a consistent and significant increase in *R*_dark_ at FG relative to Peru, with no concomitant increase in *A*_sat_ (Fig. 7). This suggests that, although taxonomy may exert appreciable control over leaf construction, and both respiratory and photosynthetic properties, leaf nutrient constraints can lead to significant variation in respiration. This result suggests that the effect of nutrient limitation on the leaf carbon balance is likely to result from substantial shifts in respiration as well as photosynthesis, with consequences for understanding and modelling the carbon balance across tropical forests.

### Taxonomic and environmental influences on *R*_dark_ and *A*

Although the South American sites shared several species and higher order taxa, AUS was taxonomically distinct, consistent with the differences in SMA slopes observed in Fig. 5. In a more formal test of taxonomic influences on our data, the mixed‐effect modelling results demonstrated that taxon is a significant source of variance (Table 6). Genetic diversity has been shown elsewhere to have a strong effect on leaf N and P concentrations and regional N : P ratios (Townsend *et al*., 2007; Fyllas *et al*., 2009; Wright *et al*., 2011; Alvarez‐Clare *et al*., 2013), and taxonomic differences may have influenced the observed differences in our leaf trait datasets. Separating genetic from environmental influences is, however, difficult in natural settings; as well as a shift in *R*_dark_ between species common to Peru and FG (Fig. 7), there was also a significantly higher LMA at FG (*P* = 0.006), a trait that is thought to be under stronger genetic than environmental control in lowland tropical forests (Fyllas *et al*., 2009).

We found that no single universal scaling relationship could account for variations in *R*_dark_ across the three different tropical sites used here. Our data support the proposal that both P and N are important predictors of *R*_dark_ across different biogeographical regions exhibiting a range of soil and leaf P values. However, contrary to our working hypotheses H1 and H2, we found that, despite a positive relationship existing between *R*_dark_ and P across all sites: (1) *R*_dark_ is not depressed at sites with lower leaf and soil P, but instead is largest at the site with the lowest available soil and leaf P (FG); and (2) that the site with the lowest leaf P has the greatest *R*_dark_ per unit N and P. These results indicate that the respiratory capacity and/or demand for respiratory products is greater at the most nutrient‐limited site, perhaps reflecting regional differences in one or more environmental factors (e.g. seasonality of rainfall) and how these differences impact on respiratory energy demand. As a consequence the *R*_dark_ : *A* ratio was elevated at the site with the lowest soil and leaf P. This result was not just a consequence of taxonomic differences between sites, as it was maintained when species common to the two South American sites were analysed independently of the main dataset. We did, however, find that phylogeny played a significant role in controlling *R*_dark_, and therefore that limitations to *R*_dark_ were likely to be the result of an interaction of environmental and genetic factors. Finally, our analysis indicated that the use of a single explanatory relationship for *R*_dark_ is not appropriate across tropical forests and is likely to produce substantial error in modelling and model‐based analyses by masking critical regional differences in physiological performance by the natural vegetation. Resolving the complexity of what drives the differences in *R*_dark_ among different tropical regions is key to understanding their functioning, particularly considering that our data suggest that nutrient limitations may be more critical for *R*_dark_ than for *A*.

## Author contributions

L.R., J.Z‐C., P.M., O.K.A., K.J.B., M.H.T., D.B. and O.L.P. planned and designed the research. L.R., J.Z‐C., P.M., O.K.A., K.J.B., B.B., N.S., E.C., D.J.M. and A.F. conducted fieldwork and analysed the data. L.R., J.Z‐C., P.M. and O.K.A. wrote the manuscript, with contributions from all authors.

## Supporting information

Please note: Wiley Blackwell are not responsible for the content or functionality of any supporting information supplied by the authors. Any queries (other than missing material) should be directed to the *New Phytologist* Central Office.

**Fig. S1** Bar plot showing the *R*_dark_ measurements made on cut and uncut branches.
**Fig. S2** Results of measurement of *R*_dark_ from 06:00 to 18:00 h on four genera in Peru and French Guiana (FG).
