# Supplementary material for: Scaling leaf respiration with nitrogen and phosphorus in tropical forests across two continents
Source: New Phytol. 2016 May 9;214(3):1064–77. doi: 10.1111/nph.13992 (PMC5412872; doi:10.1111/nph.13992)

New Phytologist

***New Phytologist* Supporting Information**

Article title: **Scaling leaf respiration with nitrogen and phosphorus in tropical forests across two continents**

Authors: Lucy Rowland, Joana Zaragoza-Castells, Keith J. Bloomfield, Matthew H. Turnbull, Damien Bonal, Benoit Burban, Norma Salinas, Eric Cosio, Daniel, J. Metcalfe, Andrew Ford, Oliver L. Phillips, Owen K. Atkin and Patrick Meir

Article acceptance date: 30 March 2016

The following Supporting Information is available for this article:

Fig. S1 Bar plot showing the R_{dark} measurements made on cut and uncut branches. Mean and standard error on uncut (solid fill) and cut (dashed lines) on three leaves from three genera from Peru (black) and French Guiana (FG; grey). No significant differences between the cut and uncut branches were found.

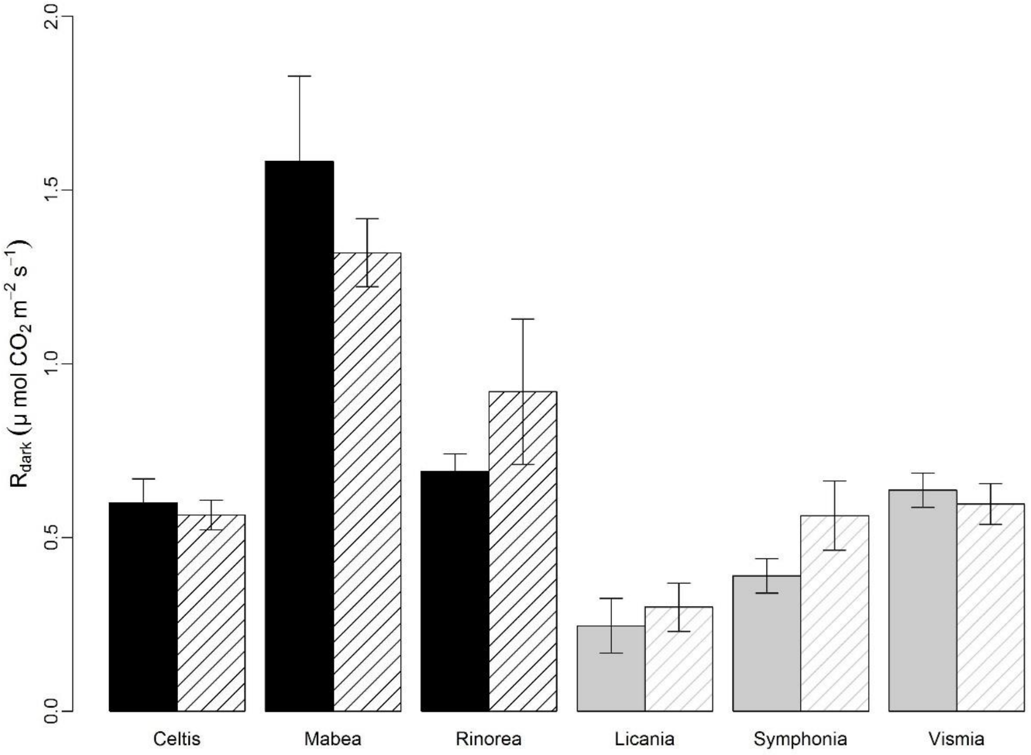

Fig. S2 Results of measuring R_{dark} from 06:00 to 18:00 h on four genera in Peru (a) and French Guiana (FG; b). Each point represents the average and standard error of R_{dark} measured on three leaves, from the same three trees per genus (*n*=9 per genus) from which a new branch was cut at 2-h intervals.

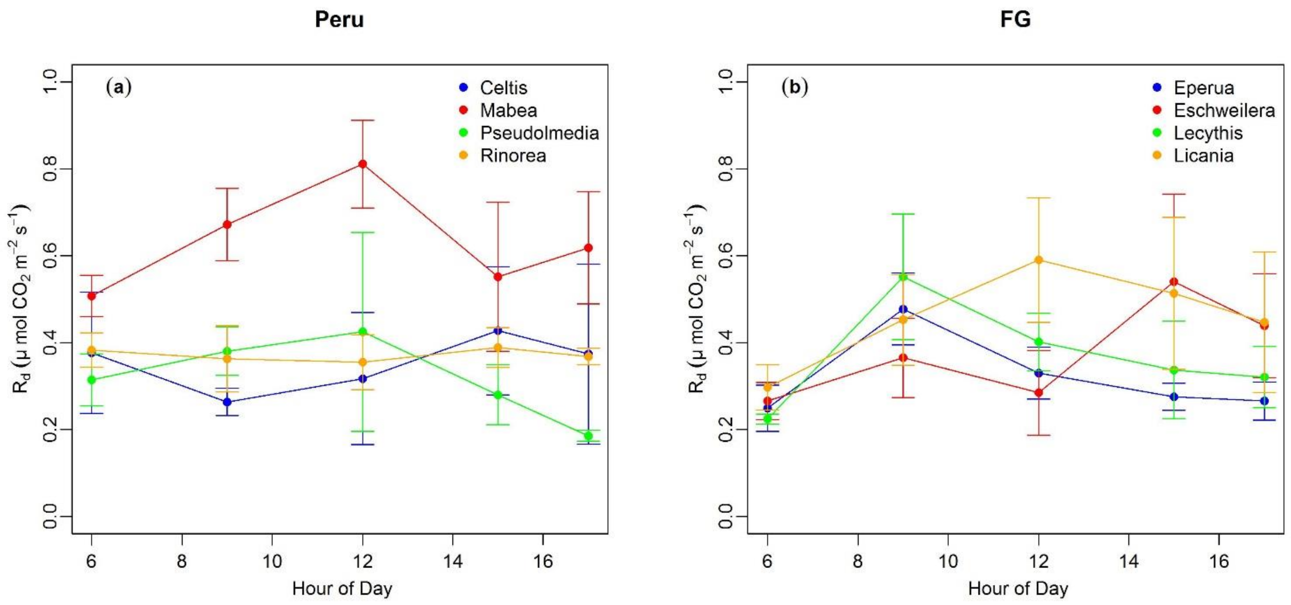

Supplement: Supplementary file 1 — Fig. S1 Bar plot showing the R dark measurements made on cut and uncut branches. Fig. S2 Results of measurement of R dark from 06:00 to 18:00 h on four genera in Peru and French Guiana (FG). [file NPH-214-1064-s001.pdf]
